# Supplementary material for: Crossing effects in the tactile temporal order judgment task: a meta-analysis
Source: Front Neurosci. 2026 Aug 10;20:1824618. doi: 10.3389/fnins.2026.1824618 (PMC13500511; doi:10.3389/fnins.2026.1824618)
Supplement: Supplementary file 1 [file Data_Sheet_1.pdf]

Supplementary Information for “Crossing effects in the tactile temporal order judgment task: A meta-analysis”

Nina Schmelter, Benedikt Langenberg, Axel Mayer, Tobias Heed

Supplement A

Correlation of uncrossed and crossed conditions

The correlation of uncrossed and crossed conditions was required for our analysis but was not reported in any study. Using a default correlation of .5 may lead to inaccuracies by overestimating the effect size if the true correlation exceeds .5 and by underestimating it if the true correlation is less than .5. We computed the correlation by utilizing openly accessible raw data obtained from studies that were part of the present meta-analysis. We accessed data from the following studies: Azañón, Mihaljevic, et al. (2016)[1], Heed et al. (2016)[2], Sharp et al. (2018)[3]. We selected subsets of the available data that closely aligned with a typical TOJ task to estimate the crossing effect (Table A1). Afterwards, we calculated a mean for the uncrossed and crossed conditions per participant and merged all data into one dataset. From this dataset the correlation between both conditions was calculated, $r(51) = .52$, $p < .001$, 95% CI [.29, .69].

Table A1. Data subset used to calculate the correlation between uncrossed and crossed conditions

Study	Data subset
Azañón, Mihaljevic, et al. (2016)[1]	Condition “Aligned”
Heed et al. (2016)[2]	Condition “All-straight”
Sharp et al. (2018)[3]	Control group

However, it is important to highlight that the correlation between both conditions varied strongly between studies, as shown in Table A2.

Table A2. Correlation coefficients for each study

Study	*r*	df	95% CI	*p*
Azañón, Mihaljevic, et al. (2016)[1]	.69	14	.29 - .88	.003
Heed et al. (2016)[2]	.09	22	-.32 - .48	.675
Sharp et al. (2018)[3]	.5	11	-.07 - .82	.083

Note. CI = confidence interval, *df* = degrees of freedom.

Furthermore, combining all the data into a single dataset assumes data independence and overlooks potential dependencies within each study. To address these limitations, we assessed the validity of the estimated correlation in two ways: First, replacing the estimate by extreme values (0.001; 0.999) in Equation 6 changed effect size estimates only minimally (Table A3). Second, computing Cohen's d_z with Equation 6 including only those studies that had reported effect sizes, *t*-, or *F*-values, too, led to only a small modulation of effect size estimates (Table A4).

Table A3. Effect sizes (Cohen's d_z) for different correlation estimates.

	Cohens d_z calculated from				
Study	*r* = .001	*r* = .09	*r* = .518	*r* = .69	*r* = .999
Azañón & Soto-Faraco (2007)[4] – exp. 1	1.02	1.03	1.08	1.1	1.14
Azañón & Soto-Faraco (2007)[4] – exp. 2	1.33	1.35	1.47	1.53	1.65

Note. Exp. = experiment

Table A4. Effect size (Cohen's d_z) comparisons for studies where t-/F-value and mean and standard deviations of the same measurement were available.

	Cohens d_z calculated from					
Study	*t*-/*F*-value	*r* = .001	*r* = .09	*r* = .518	*r* = .69	*r* = .999
Cadieux et al. (2010)[5]	2.14	1.88	1.95	2.5	2.89	4.69
Crollen, Albouy, et al. (2017)[6]	1.09	0.94	0.96	1.13	1.22	1.48

Crollen, Lazzouni, et al. (2017)[7]	0.92	0.93	0.93	0.97	0.98	1.01
Heed et al. (2012)[8]	1.72	1.7	1.76	2.17	2.45	3.38
Moseley et al. (2012)[9]	1.8	1.39	1.46	2	2.49	20.16

Supplement B

Table B1. Experiments That Did Not Meet the Inclusion Criteria: References and Reasons for Exclusion.

Study	Reason for exclusion
Azañón et al. (2010)[10] - exp. 1 - 3	Dependent Variable = Visual performance
Azañón et al. (2018)[11]	Mean age = 8.24
Azañón et al. (2015)[12] - exp. 2 & 3	Did not provide sufficient data (no main effect of crossing status reported)
Azañón et al (2015)[12] – exp. 4	Visual-tactile task
Azañón, Mihaljevic, et al. (2016)[1]– exp. 1 & 2	Hands were vertically misaligned
Azañón, Radulova, et al. (2016) [13] – exp. 1 & 2	Stimulation on shoulders and elbows
Azañón, Radulova, et al. (2016) [13] – exp. 3	Stimulation on a single skin location
Badde et al. (2017)[14] – exp. 2	Stimulation with multiple stimuli
Cadieux & Shore (2013)[15] – Eyes open, lights off	Eyes open, but light off
Cadieux & Shore (2013)[15] – exp. 1b	Eyes open/closed as within-subject factor
Cadieux & Shore (2013)[15] – exp. 2	Eyes open/closed as within-subject factor
Cadieux et al. (2010)[5] – exp. 2	No TOJ-task
Craig & Belser (2006)[16] – exp. 1 & 2	Did not provide sufficient data (only TOJ-data with training)
Craig & Belser (2006)[16] – exp. 3	Did not provide sufficient data (only comparison between control group and musicians)
Crollen, Albouy, et al. (2017)[6] – exp. 2	No TOJ-task
Di Pino et al (2020)[17]	No healthy control group
Ferri et al. (2016)[18]	Did not provide sufficient data (only time window of judgement reversal)
Heed et al. (2012)[8] – exp. 3	Crossing of fingers within one hand
Heed et al. (2016)[2] – exp. 2	Did not provide sufficient data (no main effect of crossing status reported)
Hense et al. (2019)[19] – exp. 1	Did not provide sufficient data (no data from the control group)
Hense et al. (2019)[19] – exp. 2	No TOJ-task
Manfron et al. (2021)[20]	No investigation of the crossing effect
Moharramipour & Kitazawa (2021)[21]	Did not provide sufficient data (no main effect of the crossing status)
Moharramipour et al. (2023)[22] – exp. 1 & 2	Did not provide sufficient data (no behavioral data)
Moseley et al. (2012)[9] – exp. 2 - 5	No healthy control group
Ora et al. (2016)[24]	Did not provide sufficient data (no behavioral data)

Ritterband-Rosenbaum et al. (2014)[25] – exp. 1 - 5	Degrees of freedom do not fit number of participants
Roerts & Humphreys (2008)[26] – exp. 2 & 3	No „typical“ TOJ-task
Shore et al. (2002)[27] – exp. 2	Visual stimuli
Shore et al. (2002)[27] – exp. 3	Same participants as in experiment 1
Takahashi & Kitazawa (2007)[28]	Did not provide sufficient data (only graphical report of behavioral data)
Tamè et al. (2017)[29]	Localization task of fingers of one hand
Unwalla et al. (2020)[30] – exp. 2	Did not provide sufficient data (only graphical report of crossing effect)
Wada et al. (2012)[32]	Did not provide sufficient data (only graphical report of behavioral data)
Wada et al. (2014)[33]	Mean age = 11.7
Yamamoto & Kitazawa (2001)[34]	Did not provide sufficient data (no standard deviation of flip parameters)

Note. Exp. = experiment

Table B2. Limitations of included studies.

Author	Limitation	Dependency
Azañón & Soto-Faraco (2007)[4] - exp. 1	Participants saw rubber hands (in the same position as their own hands)	Yes
Azañón & Soto-Faraco (2007)[4] - exp. 2	Participants saw rubber hands (in the same position as their own hands)	Yes
Azañón et al (2015)[12]	Main effect (posture change block wise & after 1 – 3 trials)	No
Azañón, Mihaljevic, et al. (2016)[1]	-	No
Azañón, Radulova, et al. (2016)[13]	-	No
Badde et al. (2014)[35] - exp. 1	Main effect (working memory load: no, low & high)	Yes
Badde et al. (2014)[35] - exp. 2	Main effect (working memory load: no, low & high)	Yes
Badde et al. (2015)[36] - exp. 1	Different stimulus frequency	Yes
Badde et al. (2015)[36] - exp. 2	Different stimulus frequency	Yes
Badde et al. (2017)[14]	Main effect (normal & with sound in between the stimuli)	No
Cadieux & Shore (2013)[15] - eyes closed, feet external	-	Yes
Cadieux & Shore (2013)[15] - eyes closed, hands	-	Yes
Cadieux & Shore (2013)[15] - eyes open, feet external	-	Yes
Cadieux & Shore (2013)[15] - eyes open, hands	-	Yes
Cadieux et al. (2010)[5]	-	No
Crollen, Albouy, et al. (2017)[6]	-	No
Crollen, Lazzouni, et al. (2017)[7]	Participants completed the task while lying	No
Crollen et al. (2019)[37]	-	No
Heed et al. (2012)[8] - exp. 1	Main effect (control, hands flat & turned)	Yes
Heed et al. (2012)[8] - exp. 2	-	Yes
Heed et al. (2016)[2]	Main effect (all straight, eyes to side & eyes and arms to side)	No
Kóbor et al., 2006[38]	main effect (hands in front & behind the back)	No
Landry & Champoux (2018)[39]	-	No
Moseley et al. (2012)[9]	-	No

Nishikawa et al. (2015)[23]	Values for the measure of variability were assumed to be standard deviations	No
Roberts & Humphreys (2008)[26]	Different stimulation frequency	No
Röder et al. (2004)[40] - eyes closed	-	Yes
Röder et al. (2004)[40] - eyes open	-	Yes
Sambo et al. (2013)[41]	-	No
Schicke & Röder (2006)[42]	Main effect (stimulation on hand & feet)	No
Sharp et al. (2018)[3]	-	No
Shore et al. (2002)[27]	-	No
Soto-Faraco & Azañón, 2013[43]	-	No
Unwalla et al. (2020)[30]	-	No
Unwalla, Goldreich, et al. (2021)[44] - exp. 1	-	Yes
Unwalla, Goldreich, et al. (2021)[44] - exp. 2	-	Yes
Unwalla, Cadieux, et al. (2021)[45] - eyes closed	-	Yes
Unwalla, Cadieux, et al. (2021)[45] - eyes open	-	Yes
Wada et al. (2004)[31]	Right- and left-handed participants	No

Note. Exp. = experiment

Table B3. Specifications for subgroup analysis outcome measure.

Study	Specification
Azañón et al. (2015)[12]	Logistic function to calculate JND 75%
Azañón, Mihaljevic, et al. (2016)[1]	Logit transformation
Azañón, Radulova, et al. (2016)[13]	Probit transformation
Badde et al. (2017)[14]	χ2-value from GLMM coded as accuracy
Crollen et al. (2019)[37]	Probit transformation
Crollen, Albouy, et al. (2017)[6]	Probit transformation
Crollen, Lazzouni, et al. (2017)[7]	Probit transformation
Kóbor et al. (2006)[38]	Weibull transformation
Moseley et al. (2012)[9]	Probit transformation
Roberts & Humphreys (2008)[26]	Logistic slope comparison without transformation

Röder et al. (2004)[40] - eyes closed	Probit transformation
Röder et al. (2004)[40] - eyes open	Probit transformation
Sambo et al. (2013)[41]	Not transformed Gaussian Cumulative Function
Schicke & Röder (2006)[42]	Probit transformation

Table B4 Specifications for subgroup analysis response modality

Study	Specification
Roberts & Humphreys (2008)[26]	Responses with a single foot (by lifting the toe or the heel)
Schicke & Röder (2006)[42]	Responses with hands and feet but coded as hands since participants were instructed to respond with the stimulated body part
Unwalla, Goldreich, et al. (2021)[44] - exp. 1	Selection of Feet anatomical due to the limited availability of studies with that condition
Unwalla, Goldreich, et al. (2021)[44] - exp. 2	Selection of Feet anatomical due to the limited availability of studies with that condition

Note. Exp. = experiment

Table B5 Specifications for subgroup analysis task instructions about speed

Study	Specification
Roberts & Humphreys (2008)[26]	Coded as unspeeded since accuracy was stressed rather than the speed of responding
Sambo et al. (2013)[41]	Coded as time restricted since participants were instructed to respond "as accurately and as rapidly as possible“

Supplement C

Forest Plots grouped by moderator variable

Study or Subgroup	Cohens dz	SE	Weight	Cohens dz IV, Random, 95% CI
**Analysis.method = JND 84%**				
(1) Azañón & Soto-Faraco (2007) - experiment 1	1.0784	0.3158	3.6%	1.08 [0.46; 1.70]
(2) Azañón & Soto-Faraco (2007) - experiment 2	1.4715	0.3556	3.1%	1.47 [0.77; 2.17]
(33) Soto-Faraco & Azañón (2013)	0.7888	0.2117	5.2%	0.79 [0.37; 1.20]
**Total (95% CI)**			**11.9%**	**1.04 [0.20; 1.87]**
Heterogeneity: Tau^2 = 0.0430; Chi^2 = 2.81, df = 2 (P = 0.2448); I^2 = 28.9%				
**Analysis.method = JND 75%**				
(22) Kóbor et al. (2006)	1.0878	0.3274	3.4%	1.09 [0.45; 1.73]
(24) Moseley et al. (2012)	1.8025	0.6474	1.2%	1.80 [0.53; 3.07]
(27) Röder et al. (2004) - eyes closed	1.6129	0.4957	1.9%	1.61 [0.64; 2.58]
(28) Röder et al. (2004) - eyes open	1.0567	0.4198	2.5%	1.06 [0.23; 1.88]
(29) Sambo et al. (2013)	1.5964	0.5211	1.8%	1.60 [0.57; 2.62]
(3) Azañón et al. (2015)	0.9309	0.3995	2.6%	0.93 [0.15; 1.71]
(30) Schicke & Röder (2006)	2.2107	0.7521	1.0%	2.21 [0.74; 3.68]
(4) Azañón, Mihaljevic, et al. (2016)	0.9125	0.3296	3.4%	0.91 [0.27; 1.56]
**Total (95% CI)**			**17.8%**	**1.23 [0.90; 1.56]**
Heterogeneity: Tau^2 = 0.0430; Chi^2 = 5.39, df = 7 (P = 0.6122); I^2 = 0%				
**Analysis.method = slope**				
(15) Cadieux et al. (2010)	2.1362	0.2731	4.2%	2.14 [1.60; 2.67]
(16) Crollen, Albouy, et al. (2017)	1.0885	0.4518	2.2%	1.09 [0.20; 1.97]
(17) Crollen, Lazzouni, et al. (2017)	0.9166	0.4217	2.4%	0.92 [0.09; 1.74]
(18) Crollen et al. (2019)	1.6086	0.4493	2.2%	1.61 [0.73; 2.49]
(26) Roberts & Humphreys (2008)	1.1817	0.5462	1.6%	1.18 [0.11; 2.25]
(32) Shore et al. (2002)	1.9351	0.7422	1.0%	1.94 [0.48; 3.39]
(5) Azañón, Radulova, et al. (2016)	1.1880	0.4706	2.1%	1.19 [0.27; 2.11]
**Total (95% CI)**			**15.7%**	**1.51 [1.03; 1.98]**
Heterogeneity: Tau^2 = 0.0430; Chi^2 = 9.24, df = 6 (P = 0.1605); I^2 = 35.1%				
**Analysis.method = Accuracy**				
(10) Badde et al. (2017)	0.7224	0.3082	3.7%	0.72 [0.12; 1.33]
(11) Cadieux & Shore (2013) - eyes closed, feet external	1.6101	0.5583	1.6%	1.61 [0.52; 2.70]
(12) Cadieux & Shore (2013) - eyes closed, hands	1.3014	0.3333	3.3%	1.30 [0.65; 1.95]
(13) Cadieux & Shore (2013) - eyes open, feet external	2.3970	0.7433	1.0%	2.40 [0.94; 3.85]
(14) Cadieux & Shore (2013) - eyes open, hands	1.6010	0.3733	2.9%	1.60 [0.87; 2.33]
(19) Heed et al. (2012) - experiment 1	1.4733	0.4265	2.4%	1.47 [0.64; 2.31]
(20) Heed et al. (2012) - experiment 2	1.7238	0.4916	1.9%	1.72 [0.76; 2.69]
(21) Heed et al. (2016)	1.6311	0.3384	3.3%	1.63 [0.97; 2.29]
(23) Landry & Champoux (2018)	2.4298	0.4982	1.9%	2.43 [1.45; 3.41]
(31) Sharp et al. (2018)	2.0924	0.5921	1.4%	2.09 [0.93; 3.25]
(34) Unwalla et al. (2020)	1.6435	0.3793	2.8%	1.64 [0.90; 2.39]
(35) Unwalla, Goldreich, et al. (2021) - experiment 1	1.9387	0.4223	2.4%	1.94 [1.11; 2.77]
(36) Unwalla, Goldreich, et al. (2021) - experiment 2	1.1193	0.2020	5.4%	1.12 [0.72; 1.52]
(37) Unwalla, Cadieux, et al. (2021) - eyes closed	1.3685	0.3419	3.2%	1.37 [0.70; 2.04]
(38) Unwalla, Cadieux, et al. (2021) - eyes open	1.3774	0.3431	3.2%	1.38 [0.71; 2.05]
(6) Badde et al. (2014) - experiment 1	1.5312	0.4023	2.6%	1.53 [0.74; 2.32]
(7) Badde et al. (2014) - experiment 2	2.0772	0.4913	1.9%	2.08 [1.11; 3.04]
(8) Badde et al. (2015)- experiment 1	1.7541	0.5233	1.8%	1.75 [0.73; 2.78]
(9) Badde et al. (2015)- experiment 2	1.9977	0.5437	1.7%	2.00 [0.93; 3.06]
**Total (95% CI)**			**48.5%**	**1.53 [1.33; 1.73]**
Heterogeneity: Tau^2 = 0.0430; Chi^2 = 20.58, df = 18 (P = 0.3009); I^2 = 12.6%				
**Analysis.method = A**				
(25) Nishikawa et al. (2015)	1.7000	0.3873	2.7%	1.70 [0.94; 2.46]
(39) Wada et al. (2004)	2.0493	0.3323	3.3%	2.05 [1.40; 2.70]
**Total (95% CI)**			**6.1%**	**1.89 [-0.31; 4.10]**
Heterogeneity: Tau^2 = 0.0430; Chi^2 = 0.47, df = 1 (P = 0.4937); I^2 = 0%				
**Total (95% CI)**			**100.0%**	**1.45 [1.30; 1.59]**
**Prediction interval**				**[0.88; 2.01]**

Heterogeneity: Tau^2 = 0.0715; Chi^2 = 52.38, df = 38 (P = 0.0603); I^2 = 27.5%
Residual heterogeneity: Tau^2 = 0.0430; Chi^2 = 38.50, df = 34 (P = 0.2731); I^2 = 11.7%
Test for subgroup differences: Chi^2 = 14.24, df = 4 (P = 0.0066)

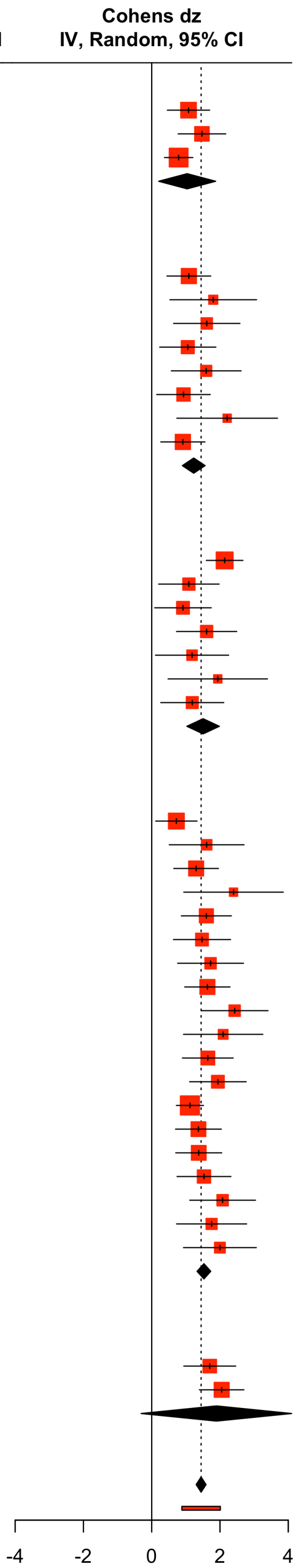

Figure C1. Forest plot of the effect sizes of included studies grouped by the moderator analysis method. *SE* = standard error, CI = confidence interval, *df* = degrees of freedom. Red squares represent the point estimates for each study and black lines are 95% confidence intervals. The size of the squares reflects the study weight with which a study contributes to the pooled effect size. The black diamond symbolizes the pooled effect estimate, while the red line below denotes the prediction interval.

Study or Subgroup	Cohens dz	SE	Weight	Cohens dz IV, Random, 95% CI
**Eyes = open**				
(1) Azañón & Soto-Faraco (2007) - experiment 1	1.0784	0.3158	4.5%	1.08 [0.46; 1.70]
(13) Cadieux & Shore (2013) - eyes open, feet external	2.3970	0.7433	1.2%	2.40 [0.94; 3.85]
(14) Cadieux & Shore (2013) - eyes open, hands	1.6010	0.3733	3.6%	1.60 [0.87; 2.33]
(15) Cadieux et al. (2010)	2.1362	0.2731	5.3%	2.14 [1.60; 2.67]
(19) Heed et al. (2012) - experiment 1	1.4733	0.4265	3.0%	1.47 [0.64; 2.31]
(2) Azañón & Soto-Faraco (2007) - experiment 2	1.4715	0.3556	3.8%	1.47 [0.77; 2.17]
(20) Heed et al. (2012) - experiment 2	1.7238	0.4916	2.4%	1.72 [0.76; 2.69]
(21) Heed et al. (2016)	1.6311	0.3384	4.1%	1.63 [0.97; 2.29]
(24) Moseley et al. (2012)	1.8025	0.6474	1.5%	1.80 [0.53; 3.07]
(28) Röder et al. (2004) - eyes open	1.0567	0.4198	3.0%	1.06 [0.23; 1.88]
(3) Azañón et al. (2015)	0.9309	0.3995	3.3%	0.93 [0.15; 1.71]
(32) Shore et al. (2002)	1.9351	0.7422	1.2%	1.94 [0.48; 3.39]
(33) Soto-Faraco & Azañón (2013)	0.7888	0.2117	6.9%	0.79 [0.37; 1.20]
(38) Unwalla, Cadieux, et al. (2021) - eyes open	1.3774	0.3431	4.0%	1.38 [0.71; 2.05]
(6) Badde et al. (2014) - experiment 1	1.5312	0.4023	3.2%	1.53 [0.74; 2.32]
(7) Badde et al. (2014) - experiment 2	2.0772	0.4913	2.4%	2.08 [1.11; 3.04]
(8) Badde et al. (2015)- experiment 1	1.7541	0.5233	2.1%	1.75 [0.73; 2.78]
(9) Badde et al. (2015)- experiment 2	1.9977	0.5437	2.0%	2.00 [0.93; 3.06]
**Total (95% CI)**			**57.5%**	**1.50 [1.28; 1.72]**
Heterogeneity: $Tau^2 = 0.0903$; $Chi^2 = 26.18$, df = 17 (P = 0.0713); $I^2 = 35.1\%$				
**Eyes = closed**				
(11) Cadieux & Shore (2013) - eyes closed, feet external	1.6101	0.5583	1.9%	1.61 [0.52; 2.70]
(12) Cadieux & Shore (2013) - eyes closed, hands	1.3014	0.3333	4.2%	1.30 [0.65; 1.95]
(16) Crollen, Albouy, et al. (2017)	1.0885	0.4518	2.7%	1.09 [0.20; 1.97]
(17) Crollen, Lazzouni, et al. (2017)	0.9166	0.4217	3.0%	0.92 [0.09; 1.74]
(18) Crollen et al. (2019)	1.6086	0.4493	2.7%	1.61 [0.73; 2.49]
(22) Kóbor et al. (2006)	1.0878	0.3274	4.3%	1.09 [0.45; 1.73]
(25) Nishikawa et al. (2015)	1.7000	0.3873	3.4%	1.70 [0.94; 2.46]
(26) Roberts & Humphreys (2008)	1.1817	0.5462	2.0%	1.18 [0.11; 2.25]
(27) Röder et al. (2004) - eyes closed	1.6129	0.4957	2.3%	1.61 [0.64; 2.58]
(29) Sambo et al. (2013)	1.5964	0.5211	2.2%	1.60 [0.57; 2.62]
(30) Schicke & Röder (2006)	2.2107	0.7521	1.1%	2.21 [0.74; 3.68]
(37) Unwalla, Cadieux, et al. (2021) - eyes closed	1.3685	0.3419	4.1%	1.37 [0.70; 2.04]
(39) Wada et al. (2004)	2.0493	0.3323	4.2%	2.05 [1.40; 2.70]
(4) Azañón, Mihaljevic, et al. (2016)	0.9125	0.3296	4.3%	0.91 [0.27; 1.56]
**Total (95% CI)**			**42.5%**	**1.39 [1.17; 1.61]**
Heterogeneity: $Tau^2 = 0.0090$; $Chi^2 = 11.4$, df = 13 (P = 0.5777); $I^2 = 0\%$				
**Total (95% CI)**			**100.0%**	**1.45 [1.30; 1.60]**
**Prediction interval**				**[0.92; 1.97]**
Heterogeneity: $Tau^2 = 0.0587$; $Chi^2 = 37.68$, df = 31 (P = 0.1901); $I^2 = 17.7\%$				
Test for subgroup differences: $Chi^2 = 0.54$, df = 1 (P = 0.4626)				

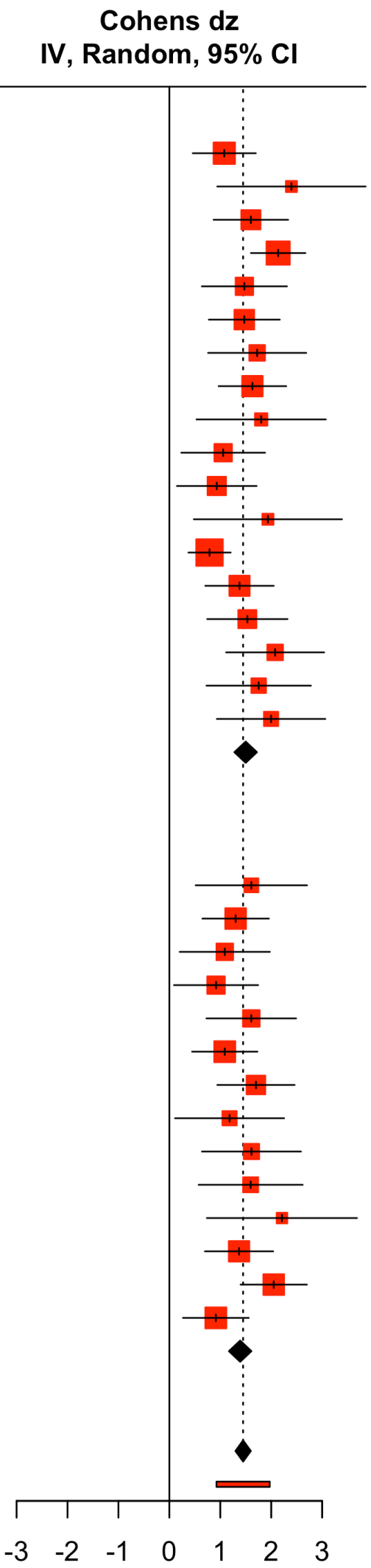

Figure C2. Forest plot of the effect sizes of included studies grouped by the moderator visual input. *SE* = standard error, CI = confidence interval, *df* = degrees of freedom. Red squares represent the point estimates for each study and black lines are 95% confidence intervals. The size of the squares reflects the study weight with which a study contributes to the pooled effect size. The black diamond symbolizes the pooled effect estimate, while the red line below denotes the prediction interval.

Study or Subgroup	Cohens dz	SE	Weight	Cohens dz IV, Random, 95% CI
**Response.modality = hands**				
(1) Azañón & Soto-Faraco (2007) - experiment 1	1.0784	0.3158	3.6%	1.08 [0.46; 1.70]
(10) Badde et al. (2017)	0.7224	0.3082	3.7%	0.72 [0.12; 1.33]
(12) Cadieux & Shore (2013) - eyes closed, hands	1.3014	0.3333	3.3%	1.30 [0.65; 1.95]
(14) Cadieux & Shore (2013) - eyes open, hands	1.6010	0.3733	2.9%	1.60 [0.87; 2.33]
(16) Crollen, Albouy, et al. (2017)	1.0885	0.4518	2.2%	1.09 [0.20; 1.97]
(17) Crollen, Lazzouni, et al. (2017)	0.9166	0.4217	2.4%	0.92 [0.09; 1.74]
(19) Heed et al. (2012) - experiment 1	1.4733	0.4265	2.4%	1.47 [0.64; 2.31]
(2) Azañón & Soto-Faraco (2007) - experiment 2	1.4715	0.3556	3.1%	1.47 [0.77; 2.17]
(20) Heed et al. (2012) - experiment 2	1.7238	0.4916	1.9%	1.72 [0.76; 2.69]
(21) Heed et al. (2016)	1.6311	0.3384	3.3%	1.63 [0.97; 2.29]
(25) Nishikawa et al. (2015)	1.7000	0.3873	2.7%	1.70 [0.94; 2.46]
(27) Röder et al. (2004) - eyes closed	1.6129	0.4957	1.9%	1.61 [0.64; 2.58]
(28) Röder et al. (2004) - eyes open	1.0567	0.4198	2.5%	1.06 [0.23; 1.88]
(29) Sambo et al. (2013)	1.5964	0.5211	1.8%	1.60 [0.57; 2.62]
(3) Azañón et al. (2015)	0.9309	0.3995	2.6%	0.93 [0.15; 1.71]
(30) Schicke & Röder (2006)	2.2107	0.7521	1.0%	2.21 [0.74; 3.68]
(32) Shore et al. (2002)	1.9351	0.7422	1.0%	1.94 [0.48; 3.39]
(33) Soto-Faraco & Azañón (2013)	0.7888	0.2117	5.2%	0.79 [0.37; 1.20]
(34) Unwalla et al. (2020)	1.6435	0.3793	2.8%	1.64 [0.90; 2.39]
(37) Unwalla, Cadieux, et al. (2021) - eyes closed	1.3685	0.3419	3.2%	1.37 [0.70; 2.04]
(38) Unwalla, Cadieux, et al. (2021) - eyes open	1.3774	0.3431	3.2%	1.38 [0.71; 2.05]
(39) Wada et al. (2004)	2.0493	0.3323	3.3%	2.05 [1.40; 2.70]
(4) Azañón, Mihaljevic, et al. (2016)	0.9125	0.3296	3.4%	0.91 [0.27; 1.56]
(5) Azañón, Radulova, et al. (2016)	1.1880	0.4706	2.1%	1.19 [0.27; 2.11]
(6) Badde et al. (2014) - experiment 1	1.5312	0.4023	2.6%	1.53 [0.74; 2.32]
(7) Badde et al. (2014) - experiment 2	2.0772	0.4913	1.9%	2.08 [1.11; 3.04]
(8) Badde et al. (2015)- experiment 1	1.7541	0.5233	1.8%	1.75 [0.73; 2.78]
(9) Badde et al. (2015)- experiment 2	1.9977	0.5437	1.7%	2.00 [0.93; 3.06]
**Total (95% CI)**			**73.5%**	**1.36 [1.20; 1.52]**
Heterogeneity: $Tau^2 = 0.0399$; $Chi^2 = 31.32$, df = 27 (P = 0.2581); $I^2 = 13.8\%$				
**Response.modality = feet external**				
(11) Cadieux & Shore (2013) - eyes closed, feet external	1.6101	0.5583	1.6%	1.61 [0.52; 2.70]
(13) Cadieux & Shore (2013) - eyes open, feet external	2.3970	0.7433	1.0%	2.40 [0.94; 3.85]
(15) Cadieux et al. (2010)	2.1362	0.2731	4.2%	2.14 [1.60; 2.67]
(18) Crollen et al. (2019)	1.6086	0.4493	2.2%	1.61 [0.73; 2.49]
(23) Landry & Champoux (2018)	2.4298	0.4982	1.9%	2.43 [1.45; 3.41]
(26) Roberts & Humphreys (2008)	1.1817	0.5462	1.6%	1.18 [0.11; 2.25]
(31) Sharp et al. (2018)	2.0924	0.5921	1.4%	2.09 [0.93; 3.25]
**Total (95% CI)**			**14.0%**	**1.94 [1.55; 2.33]**
Heterogeneity: $Tau^2 = 0.0399$; $Chi^2 = 4.74$, df = 6 (P = 0.5780); $I^2 = 0\%$				
**Response.modality = feet anatomical**				
(22) Kóbor et al. (2006)	1.0878	0.3274	3.4%	1.09 [0.45; 1.73]
(35) Unwalla, Goldreich, et al. (2021) - experiment 1	1.9387	0.4223	2.4%	1.94 [1.11; 2.77]
(36) Unwalla, Goldreich, et al. (2021) - experiment 2	1.1193	0.2020	5.4%	1.12 [0.72; 1.52]
**Total (95% CI)**			**11.3%**	**1.27 [0.27; 2.27]**
Heterogeneity: $Tau^2 = 0.0399$; $Chi^2 = 3.3$, df = 2 (P = 0.1916); $I^2 = 39.5\%$				
**Response.modality = verbal**				
(24) Moseley et al. (2012)	1.8025	0.6474	1.2%	1.80 [0.53; 3.07]
**Total (95% CI)**			**100.0%**	**1.45 [1.30; 1.59]**
**Prediction interval**				**[0.88; 2.01]**

Heterogeneity: $Tau^2 = 0.0715$; $Chi^2 = 52.38$, df = 38 (P = 0.0603); $I^2 = 27.5\%$
Residual heterogeneity: $Tau^2 = 0.0399$; $Chi^2 = 39.37$, df = 35 (P = 0.2808); $I^2 = 11.1\%$
Test for subgroup differences: $Chi^2 = 11.68$, df = 3 (P = 0.0086)

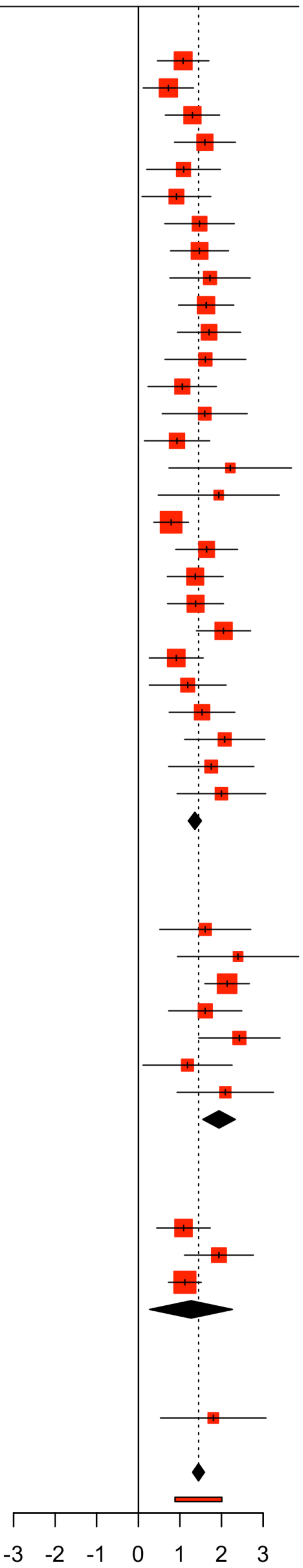

Figure C3. Forest plot of the effect sizes of included studies grouped by the moderator response modality. *SE* = standard error, CI = confidence interval, *df* = degrees of freedom. Red squares represent the point estimates for each study and black lines are 95% confidence intervals. The size of the squares reflects the study weight with which a study contributes to the pooled effect size. The black diamond symbolizes the pooled effect estimate, while the red line below denotes the prediction interval.

Study or Subgroup	Cohens dz	SE	Weight	Cohens dz IV, Random, 95% CI
**Speed = unspeeded**				
(1) Azañón & Soto-Faraco (2007) - experiment 1	1.0784	0.3158	4.0%	1.08 [0.46; 1.70]
(19) Heed et al. (2012) - experiment 1	1.4733	0.4265	2.6%	1.47 [0.64; 2.31]
(2) Azañón & Soto-Faraco (2007) - experiment 2	1.4715	0.3556	3.4%	1.47 [0.77; 2.17]
(20) Heed et al. (2012) - experiment 2	1.7238	0.4916	2.1%	1.72 [0.76; 2.69]
(22) Kóbor et al. (2006)	1.0878	0.3274	3.8%	1.09 [0.45; 1.73]
(23) Landry & Champoux (2018)	2.4298	0.4982	2.0%	2.43 [1.45; 3.41]
(26) Roberts & Humphreys (2008)	1.1817	0.5462	1.7%	1.18 [0.11; 2.25]
(27) Röder et al. (2004) - eyes closed	1.6129	0.4957	2.1%	1.61 [0.64; 2.58]
(28) Röder et al. (2004) - eyes open	1.0567	0.4198	2.7%	1.06 [0.23; 1.88]
(3) Azañón et al. (2015)	0.9309	0.3995	2.9%	0.93 [0.15; 1.71]
(30) Schicke & Röder (2006)	2.2107	0.7521	1.0%	2.21 [0.74; 3.68]
(31) Sharp et al. (2018)	2.0924	0.5921	1.5%	2.09 [0.93; 3.25]
(32) Shore et al. (2002)	1.9351	0.7422	1.0%	1.94 [0.48; 3.39]
(4) Azañón, Mihaljevic, et al. (2016)	0.9125	0.3296	3.8%	0.91 [0.27; 1.56]
(5) Azañón, Radulova, et al. (2016)	1.1880	0.4706	2.2%	1.19 [0.27; 2.11]
(8) Badde et al. (2015)- experiment 1	1.7541	0.5233	1.9%	1.75 [0.73; 2.78]
(9) Badde et al. (2015)- experiment 2	1.9977	0.5437	1.8%	2.00 [0.93; 3.06]
**Total (95% CI)**			**40.7%**	**1.38 [1.15; 1.60]**
Heterogeneity: $Tau^2 = 0.0021$; $Chi^2 = 16.17$, df = 16 (P = 0.4414); $I^2 = 1\%$				
**Speed = time restriction**				
(10) Badde et al. (2017)	0.7224	0.3082	4.2%	0.72 [0.12; 1.33]
(11) Cadieux & Shore (2013) - eyes closed, feet external	1.6101	0.5583	1.7%	1.61 [0.52; 2.70]
(12) Cadieux & Shore (2013) - eyes closed, hands	1.3014	0.3333	3.8%	1.30 [0.65; 1.95]
(13) Cadieux & Shore (2013) - eyes open, feet external	2.3970	0.7433	1.0%	2.40 [0.94; 3.85]
(14) Cadieux & Shore (2013) - eyes open, hands	1.6010	0.3733	3.2%	1.60 [0.87; 2.33]
(15) Cadieux et al. (2010)	2.1362	0.2731	4.8%	2.14 [1.60; 2.67]
(16) Crollen, Albouy, et al. (2017)	1.0885	0.4518	2.4%	1.09 [0.20; 1.97]
(17) Crollen, Lazzouni, et al. (2017)	0.9166	0.4217	2.7%	0.92 [0.09; 1.74]
(18) Crollen et al. (2019)	1.6086	0.4493	2.4%	1.61 [0.73; 2.49]
(25) Nishikawa et al. (2015)	1.7000	0.3873	3.0%	1.70 [0.94; 2.46]
(29) Sambo et al. (2013)	1.5964	0.5211	1.9%	1.60 [0.57; 2.62]
(34) Unwalla et al. (2020)	1.6435	0.3793	3.1%	1.64 [0.90; 2.39]
(35) Unwalla, Goldreich, et al. (2021) - experiment 1	1.9387	0.4223	2.7%	1.94 [1.11; 2.77]
(36) Unwalla, Goldreich, et al. (2021) - experiment 2	1.1193	0.2020	6.6%	1.12 [0.72; 1.52]
(37) Unwalla, Cadieux, et al. (2021) - eyes closed	1.3685	0.3419	3.6%	1.37 [0.70; 2.04]
(38) Unwalla, Cadieux, et al. (2021) - eyes open	1.3774	0.3431	3.6%	1.38 [0.71; 2.05]
(39) Wada et al. (2004)	2.0493	0.3323	3.8%	2.05 [1.40; 2.70]
(6) Badde et al. (2014) - experiment 1	1.5312	0.4023	2.9%	1.53 [0.74; 2.32]
(7) Badde et al. (2014) - experiment 2	2.0772	0.4913	2.1%	2.08 [1.11; 3.04]
**Total (95% CI)**			**59.3%**	**1.51 [1.31; 1.72]**
Heterogeneity: $Tau^2 = 0.0715$; $Chi^2 = 25.95$, df = 18 (P = 0.1009); $I^2 = 30.6\%$				
**Total (95% CI)**			**100.0%**	**1.47 [1.32; 1.62]**
**Prediction interval**				**[0.98; 1.96]**
Heterogeneity: $Tau^2 = 0.0526$; $Chi^2 = 42.77$, df = 35 (P = 0.1721); $I^2 = 18.2\%$				
Test for subgroup differences: $Chi^2 = 0.89$, df = 1 (P = 0.3463)				

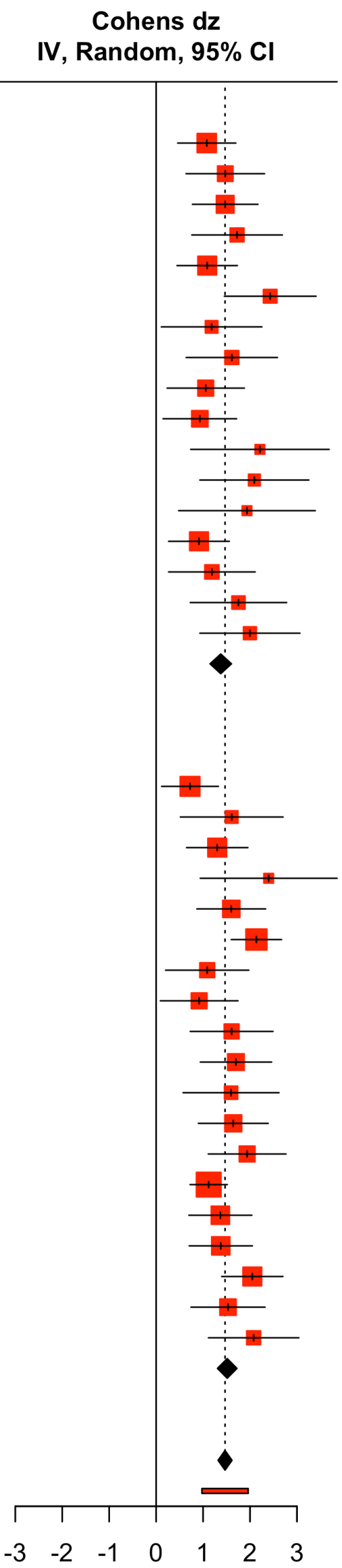

Figure C4. Forest plot of the effect sizes of included studies grouped by the moderator task instructions of speed. *SE* = standard error, CI = confidence interval, *df* = degrees of freedom. Red squares represent the point estimates for each study and black lines are 95% confidence intervals. The size of the squares reflects the study weight with which a study contributes to the pooled effect size. The black diamond symbolizes the pooled effect estimate, while the red line below denotes the prediction interval.

References

1. Azañón, E., Mihaljevic, K. & Longo, M. R. A three-dimensional spatial characterization of the crossed-hands deficit. *Cognition* **157**, 289–295 (2016).
2. Heed, T., Backhaus, J., Röder, B. & Badde, S. Disentangling the external reference frames relevant to tactile localization. *PLoS ONE* **11**, (2016).
3. Sharp, A., Landry, S. P., Maheu, M. & Champoux, F. Deafness alters the spatial mapping of touch. *PLoS ONE* **13**, e0192993 (2018).
4. Azañón, E. & Soto-Faraco, S. Alleviating the 'crossed-hands' deficit by seeing uncrossed rubber hands. *Exp. Brain Res.* **182**, 537–548 (2007).
5. Cadieux, M. L., Barnett-Cowan, M. & Shore, D. Crossing the hands is more confusing for females than males. *Experimental brain research,* **204**, 431–446 (2010).
6. Crollen, V., Albouy, F., Lepore, F. & Collignon, O. How visual experience impacts the internal and external spatial mapping of sensorimotor functions. *Scientific reports* **7**, 1022 (2017).
7. Crollen, V. *et al.* Visual experience shapes the neural networks remapping touch into external space. *Journal of neuroscience* **37**, 10097–10103 (2017).
8. Heed, T., Backhaus, J. & Roder, B. Integration of hand and finger location in external spatial coordinates for tactile localization. *Journal of Experimental Psychology: Human Perception and Performance* **38**, 386–401 (2012).
9. Moseley, G. L., Gallace, A. & Iannetti, G. D. Spatially defined modulation of skin temperature and hand ownership of both hands in patients with unilateral complex regional pain syndrome. *Brain* **135**, 3676–3686 (2012).
10. Azañón, E., Camacho, K. & Soto-Faraco, S. Tactile remapping beyond space. *European Journal of Neuroscience* **31**, 1858–1867 (2010).

11. Azañón, E., Camacho, K., Morales, M. & Longo, M. The sensitive period for tactile remapping does not include early infancy. *Child development* **89**, 1394–1404 (2018).
12. Azañón, E., Stenner, M., Cardini, F. & Haggard, P. Dynamic tuning of tactile localization to body posture. *Current Biology* **25**, 512–517 (2015).
13. Azañón, E., Radulova, S., Haggard, P. & Longo, M. R. Does the crossed-limb deficit affect the uncrossed portions of limbs? *Journal of Experimental Psychology: Human Perception and Performance* **42**, 1320–1331 (2016).
14. Badde, S., Röder, B. & Bruns, P. Task-irrelevant sounds influence both temporal order and apparent-motion judgments about tactile stimuli applied to crossed and uncrossed hands. *Attention, Perception, & Psychophysics* **80**, 773–783 (2017).
15. Cadieux, M. L. & Shore, D. I. Response demands and blindfolding in the crossed-hands deficit: an exploration of reference frame conflict. *Multisens Res* **26**, 465–482 (2013).
16. Craig, J. C. & Belser, A. N. The crossed-hands deficit in tactile temporal-order judgments: The effect of training. *Perception* **35**, 1561–1572 (2006).
17. Di Pino, G. *et al.* Sensory- and action-oriented embodiment of neurally-interfaced robotic hand prostheses. *Frontiers in Neuroscience* **14**, (2020).
18. Ferri, F., Ambrosini, E. & Costantini, M. Spatiotemporal processing of somatosensory stimuli in schizotypy. *Scientific reports* **6**, 38735 (2016).
19. Hense, M., Badde, S., Köhne, S., Dziobek, I. & Röder, B. Visual and proprioceptive influences on tactile spatial processing in adults with autism spectrum disorders. *Autism Res.* **12**, 1745–1757 (2019).

20. Manfron, L., Vanderclausen, C. & Legrain, V. No evidence for an effect of the distance between the hands on tactile temporal order judgments. *Perception* **50**, 294–307 (2021).
21. Moharramipour, A. & Kitazawa, S. What underlies a greater reversal in tactile temporal order judgment when the hands are crossed? A structural MRI study. *Cerebral Cortex Communications* **2**, (2021).
22. Moharramipour, A., Takahashi, T. & Kitazawa, S. Distinctive modes of cortical communications in tactile temporal order judgment. *Cerebral Cortex* **33**, 2982–2996 (2023).
23. Nishikawa, N., Shimo, Y., Wada, M., Hattori, N. & Kitazawa, S. Effects of aging and idiopathic Parkinson's disease on tactile temporal order judgment. *PLoS ONE* **10**, (2015).
24. Ora, H., Wada, M., Salat, D. & Kansaku, K. Arm crossing updates brain functional connectivity of the left posterior parietal cortex. *Sci Rep* **6**, 28105 (2016).
25. Ritterband-Rosenbaum, A., Hermosillo, R., Kroliczak, G. & van Donkelaar, P. Hand position-dependent modulation of errors in vibrotactile temporal order judgments:the effects of transcranial magnetic stimulation to the human posterior parietal cortex. *Experimental Brain Research* **232**, 1689–1698 (2014).
26. Roberts, R. D. & Humphreys, G. W. Task effects on tactile temporal order judgments: when space does and does not matter. *J. Exp. Psychol. Hum. Percept. Perform.* **34**, 592–604 (2008).
27. Shore, D., Spry, E. & Spence, C. Confusing the mind by crossing the hands. *Cognitive Brain Research* **14**, 153–163 (2002).

28. Takahashi, T. & Kitazawa, S. Modulation of illusory reversal in tactile temporal order by the phase of posterior α rhythm. *J. Neurosci.* **37**, 5298–5308 (2017).
29. Tamè, L., Wuhle, A., Petri, C., Pavani, F. & Braun, C. Concurrent use of somatotopic and external reference frames in a tactile mislocalization task. *Brain and cognition* **111**, 25–33 (2017).
30. Unwalla, K., Kearney, H. & Shore, D. I. Reliability of the crossed-hands deficit in tactile temporal order judgements. *Multisens Res* **34**, 387–421 (2020).
31. Wada, M., Yamamoto, S. & Kitazawa, S. Effects of handedness on tactile temporal order judgment. *Neuropsychologia* **42**, 1887–1895 (2004).
32. Wada, M. *et al.* Spatio-temporal updating in the left posterior parietal cortex. *PLoS ONE* **7**, (2012).
33. Wada, M. *et al.* Spatio-temporal processing of tactile stimuli in autistic children. *Sci Rep* **4**, 5985 (2014).
34. Yamamoto, S. & Kitazawa, S. Reversal of subjective temporal order due to arm crossing. *Nat. Neurosci.* **4**, 759–765 (2001).
35. Badde, S., Heed, T. & Röder, B. Processing load impairs coordinate integration for the localization of touch. *Atten Percept Psychophys* **76**, 1136–1150 (2014).
36. Badde, S., Röder, B. & Heed, T. Flexibly weighted integration of tactile reference frames. *Neuropsychologia* **70**, 367–374 (2015).
37. Crollen, V., Spruyt, T., Mahau, P., Bottini, R. & Collignon, O. How visual experience and task context modulate the use of internal and external spatial coordinate for perception and action. *Journal of Experimental Psychology: Human Perception and Performance* **45**, 354–362 (2019).

38. Kóbor, I., Füredi, L., Kovács, G., Spence, C. & Vidnyánszky, Z. Back-to-front: Improved tactile discrimination performance in the space you cannot see. *Neurosci. Lett.* **400**, 163–167 (2006).

39. Landry, S. P. & Champoux, F. Long-term musical training alters tactile temporal-order judgment. *Multisens Res* **31**, 373–389 (2018).

40. Röder, B., Rösler, F. & Spence, C. Early vision impairs tactile perception in the blind. *Curr. Biol.* **14**, 121–124 (2004).

41. Sambo, C. F., Gallace, A., Liang, M., Moseley, G. L. & Iannetti, G. D. The temporal order judgement of tactile and nociceptive stimuli is impaired by crossing the hands over the body midline. *Pain* **154**, 242–247 (2013).

42. Schicke, T. & Röder, B. Spatial remapping of touch: confusion of perceived stimulus order across hand and foot. *Proc Natl Acad Sci U S A* **103**, 11808–11813 (2006).

43. Soto-Faraco, S., 1970- & Azañón, E. Electrophysiological correlates of tactile remapping. *Neuropsychologia* **51**, 1584–1594 (2013).

44. Unwalla, K., Goldreich, D. & Shore, D. I. Exploring reference frame integration using response demands in a tactile temporal-order judgement task. *Multisens Res* **34**, 1–32 (2021).

45. Unwalla, K., Cadieux, M. L. & Shore, D. I. Haptic awareness changes when lying down. *Scientific reports* **11**, 13479 (2021).
