# Supplementary material for: Crossing effects in the tactile temporal order judgment task: a meta-analysis
Source: Front Neurosci. 2026 Aug 10;20:1824618. doi: 10.3389/fnins.2026.1824618 (PMC13500511; doi:10.3389/fnins.2026.1824618)
Supplement: Supplementary file 2 [file Data_Sheet_2.pdf]

Appendices for:
Crossing effects in the tactile temporal order judgment task: A meta-analysis

Nina Schmelter[1], Benedikt Langenberg[2], Axel Mayer[1], Tobias Heed[3,4]

[1] Department of Psychology, Faculty of Psychology and Sports Science, Bielefeld University, Bielefeld, Germany

[2] Department of Methodology and Statistics, Maastricht University, Netherlands

[3] Department of Psychology, University of Salzburg, Salzburg, Austria

[4] Centre for Cognitive Neuroscience, University of Salzburg, Salzburg, Austria

Appendix A

Table A1. Search String for Each Database

Database	Search string
BASE	(tactile OR hand OR arm) temporal order judg* doctype:121; english manually selected
EMBASE	('tactile temporal order' OR ((hand OR tactile OR arm) AND temporal AND order AND judg*)) AND ([article]/lim OR [article in press]/lim) AND [english]/lim
PUBMED	(tactile OR hand OR arm) AND temporal order judg* AND (english[Filter]) NOT(review pt])
Web of Science	ALL=((hand OR tactile OR arm) temporal order judg*); articles manually selected

Appendix B

Transformation of noncentral t variance to variance of Cohen's d_z

To calculate the standard error of Cohen's d_z, typically either the standard deviation of the mean difference or the correlation between the two measurements (crossed and uncrossed conditions) is needed [29,30]. However, these data were not available in all studies. Therefore, to avoid using an estimated correlation for the standard error calculation, the relationship between t and Cohen's d_z was utilized to calculate the standard errors of the effect sizes:

1. Variance of the random variable t which follows a non-central t-distribution [84] (using an approximation [85,86]):

$$Var(t) = \frac{v(1+\mu^2)}{v-2} - \frac{\mu^2 * v}{2} * \left(1 - \frac{3}{4 * v - 1}\right)^{-2} \quad \text{(B1)}$$

   where v is the degrees of freedom and μ is the noncentrality parameter.

2. Given the relation between t and Cohen's d_z [30], t can be calculated from d_z, and the sample size:

$$Cohen's\ d_z = \frac{t}{\sqrt{n}} \text{ and therefore } t = d_z * \sqrt{n} \quad \text{(B2)}$$

3. Substitution of v and μ for within-subject designs: where v = n-1 and $\mu = t = d_z * \sqrt{n}$:

$$Var(t) = \frac{(n-1)\left(1 + (d_z * \sqrt{n})^2\right)}{(n-1)-2} - \frac{(d_z * \sqrt{n})^2 * (n-1)}{2} * \left(1 - \frac{3}{4 * (n-1) - 1}\right)^{-2} \quad \text{(B3)}$$

4. Given equation (B2) the relationship between $Var(t)$ and $Var(d_z)$ can be expressed as:

$$Var(t) = n * Var(d_z) \quad \text{(B4)}$$

   therefore:

$$Var(d_z) = \frac{Var(t)}{n} \quad \text{(B5)}$$

5. Substitution of $Var(t)$ into the equation for $Var(d_z)$:

$$Var(d_z) = \frac{1}{n} * \left(\frac{(n-1)\left(1 + (d_z * \sqrt{n})^2\right)}{(n-1)-2} - \frac{(d_z * \sqrt{n})^2 * (n-1)}{2} * \left(1 - \frac{3}{4*(n-1)-1}\right)^{-2} \right) \tag{B6}$$

6. Simplified to:

$$Var(d_z) = \frac{1}{n} * \frac{n-1}{(n-1)-2} + d_z^2 * \left(\frac{n-1}{(n-1)-2} - \frac{1}{\left(1 - \frac{3}{4*(n-1)-1}\right)^2} \right) \tag{B7}$$

7. Derive the standard error SE(d_z), which in this case equals to the standard deviation and is therefore defined as the square root of the variance:

$$SE(d_z) = \sqrt{\frac{1}{n} * \frac{n-1}{(n-1)-2} + d_z^2 * \left(\frac{n-1}{(n-1)-2} - \frac{1}{\left(1 - \frac{3}{4*(n-1)-1}\right)^2} \right)} \tag{B8}$$
